# Metabolite transport across central nervous system barriers

**DOI:** 10.1177/0271678X241241908

**Published:** 2024-03-28

**Authors:** Gesa Carstens, Marcel M Verbeek, Ursula K Rohlwink, Anthony A Figaji, Lindsey te Brake, Arjan van Laarhoven

**Affiliations:** 1 Department of Internal Medicine and Radboud Center of Infectious Diseases (RCI), Radboud University Medical Center, Nijmegen, Netherlands; 2 Departments of Neurology and Human Genetics, Radboud University Medical Center, Donders Institute for Brain, Cognition, and Behavior, Nijmegen, Netherlands; 3 Division of Neurosurgery, Department of Surgery, Neuroscience Institute, 37716 University of Cape Town, Cape Town, South Africa; 4 Department of Pharmacy, Radboud University Medical Center, Nijmegen, The Netherlands

**Keywords:** Blood-brain barrier, blood-CSF barrier, brain metabolism, metabolomics, transport mechanisms

## Abstract

Metabolomic analysis of cerebrospinal fluid (CSF) is used to improve diagnostics and pathophysiological understanding of neurological diseases. Alterations in CSF metabolite levels can partly be attributed to changes in brain metabolism, but relevant transport processes influencing CSF metabolite concentrations should be considered. The entry of molecules including metabolites into the central nervous system (CNS), is tightly controlled by the blood-brain, blood-CSF, and blood-spinal cord barriers, where aquaporins and membrane-bound carrier proteins regulate influx and efflux via passive and active transport processes. This report therefore provides reference for future CSF metabolomic work, by providing a detailed summary of the current knowledge on the location and function of the involved transporters and routing of metabolites from blood to CSF and from CSF to blood.

## Introduction

The brain is widely considered the most complex organ of our body. Its incompletely understood metabolism can be dysregulated in many common neurological disorders, such as traumatic brain injury, meningitis, stroke, and neurodegeneration, which may have important implications for clinical care and the development of new therapies. Complete understanding of these disrupted processes, however, is limited by their complexity, dynamic changes over time, and the methodological difficulties in studying the brain. While brain tissue is not easily accessible, the surrounding cerebrospinal fluid (CSF) is often used as a go-to sample for metabolic studies, which have provided insights in the pathophysiology of Parkinson’s and Alzheimer’s disease,^1–3^ multiple sclerosis,^4^ tuberculous meningitis,^5,6^ and inborn errors of metabolism.^7–9^ Changes in CSF metabolites are attributed to changes in brain metabolism, but their source and transport need to be accounted for. Brain-derived metabolites include intermediate or end products of metabolic processes that occur in the cells of the CNS. In contrast, blood-derived metabolites are transported directly from the systemic metabolism without influence of CNS metabolism. All metabolites ultimately derive from dietary intake and gut bacterial modification, and after circulation through the blood compartment, they reach the CSF after transport across three barriers.

First, metabolites can enter the CNS across the blood-brain barrier (BBB), after which they reach the brain parenchyma. After metabolism by CNS cells, they diffuse as brain-derived metabolites passively into the CSF or cerebral interstitial fluid.^10^ The BBB is made up of non-fenestrated brain capillary endothelial cells, the basement membrane, pericytes, and the end-feet of astrocytes surrounding the capillaries. Tight junctions and adherens junctions connect the endothelial cells of the BBB and limit the paracellular diffusion of molecules between cells.^11^ Therefore, especially for hydrophilic and polar molecules, carrier-mediated or vesicular transport is needed to move molecules through endothelial cells.

Second, it is less recognized that CSF metabolites can also enter the spinal part of the CNS from the blood or exit to blood across the vessels that comprise the blood spinal cord barrier (BSCB). The BSCB resembles the BBB morphologically, but is probably more permeable, given the lower density of specific tight and adherence junction-associated proteins.^12,13^ During inflammatory conditions, all the above relationships may be disturbed by barrier leakage.^10,14^

Lastly, metabolites can enter the CSF directly across the blood-CSF (BCSFB) barrier located at the choroidal vessels in the ventricular system of the brain, where CSF is continuously secreted by the cells of the choroid plexus at a rate of 0.3–0.4 ml/minute in adult humans,^15^ leading to a daily production of 400–600 ml. Likewise, the total volume at a given moment shows variation, often cited as 140 ml^16^ on average, but recent MRI studies indicate that volume may be twice as large.^17^ The BCSFB encompasses fenestrated capillaries and a surrounding layer of epithelial cells closely connected by tight junctions, through which the plasma is filtered. This was previously explained using a bulk flow (unidirectional) theory, but most likely pulsatile back and forth movement promotes the exchange between capillaries and between the CSF and interstitial fluid.^15^ Additional CSF flows across the ventricular ependyma from the CNS interstitial fluid surrounding the brain parenchyma and capillaries.^18^ The BCSFB plays an important regulatory role in limiting access of plasma proteins, cells, xenobiotics, and metabolites to the CSF. Consequently, most metabolites have lower levels in CSF than in blood.^5,16^ Interestingly, the choroid plexus also enables reverse transport, removing metabolic waste products from the CSF into the blood circulation.^19^

Three pathways for CSF drainage have been proposed and currently it is under debate how their relative importance needs to be viewed (extensively reviewed in^17,20^). First, arachnoid villi were thought to play a main role. These small protrusions extending from the arachnoid mater into dural venous sinuses, allow CSF drainage through a hydrostatic pressure mechanism. Second, lymphatic drainage from the olfactory bulb, along cranial and spinal nerves and in the dura mater to lymph nodes are involved, making use of perineural sheaths. Third, the glymphatic system is the communication of subarachnoidal CSF with the perivascular space, in which bidirectional flow across endothelial barriers is proposed, driven by osmotic and hydrostatic forces.

The concentration of CSF metabolite levels therefore is the net result of 1) influx to the CSF through the BCSFB and BSCB, 2) influx of metabolites that enter the CNS via the BBB and are metabolized there before diffusing to the CSF, and 3) efflux out of the CSF.^21^ To understand metabolite transport, we can learn from studies on CNS drug delivery.^22,23^ For proteins in CSF, it is estimated that the majority is blood-derived, adding up to 80% of the total protein concentration, the majority of which is albumin.^24^ Only a minority of CSF proteins, such as S100B and neuron-specific enolase,^25^ are known to be brain-derived, but for CSF metabolites it is unknown whether they are blood or brain-derived. CNS metabolite composition is important for neuronal function, and therefore transport mechanisms are tightly regulated. To understand disrupted cerebral metabolism in disease and interpret studies that report these disruptions, it is critical to understand these transport mechanisms. In this manuscript we therefore aim to comprehensively review available passive and active routes of diffusion and transport mechanisms of metabolites into the CSF, considering the location and directionality of transport at the BBB and the BCSFB. We reviewed transport mechanisms and transporter superfamilies important for the transport of metabolites. Of those mechanisms and transporter superfamilies, we reviewed individual transporters of metabolites, but also those of water, electrolytes or proteins for completeness. Finally, we review the strategies that could be employed to use the CSF metabolome to understand what happens in brain metabolism in health and disease.

## Methods

The references for this review were extracted by searches of PubMed between 1960 and March 2023 using the search terms “blood-brain barrier” or “BBB” or “blood-CSF barrier” or “choroid plexus” in combination with the different transporter superfamilies and families “aquaporin”, “ABC transporter”, “P-glycoprotein”, “multidrug resistance protein”, “breast cancer-related protein”, “solute carrier”, “SLC transporter”, “organic anion transporting polypeptide”, “organic anion transporter”, “organic cation transporter”, “glucose transporter”, “proton-coupled oligopeptide transporter”, and “amino acid transporter”. Additionally, references from relevant articles were used.

Despite the close relationship between RNA and protein, it has become increasingly clear that protein abundance cannot be directly inferred from corresponding mRNA abundance as regulatory processes^26,27^ and post-transcriptional mechanisms determine protein levels independently of mRNA abundance.^28^ Moreover, protein-based methods are known for their high specificity, ensuring that the identified transporters are indeed present within the barriers of humans ensures accurately characterizing the transport proteins involved in the movement of metabolites. Therefore, transporters were only included if the presence of transporters were identified in humans using protein-based methods. The location of the transporters in Table 1 was assessed using the location by immunohistochemistry in human or animal studies or indicated as ‘unknown’ when its existence was detected by proteomics or Western blot without proof of its exact location. In case of transporters capable of bidirectional transport, i.e., for solute carriers, the net flux over the transporter is additionally indicated when known. Transporters found to be present by a protein-based technique, but for which location and directionality are not known are indicated in the table but not in the figure. Hypothetical transporters however, i.e., those exclusively identified with RNA-based method such as quantitative PCR, micro-array, or RNA sequencing, but without protein-based proof, are beyond the scope of this review. Of note, metabolites, i.e. products or intermediates derived from metabolic processes, are referred to as “substrates” in relation to their transport by specific transporters.

**Table 1.** Overview of channels, transporters and receptors involved in transport across the human blood-brain barrier and blood-CSF barrier based on protein-based detection methods.

	Alias/Human gene name	Blood-brain barrier			Blood-CSF barrier			
Transporter		Cellular location^a^	Directionality^b^	References	Cellular location^c^	Directionality^d^	References	Transported^e^ molecules
Vesicular transport								
LRP1, LRP2	LRP1, LRP2	Unknown (h)	Bidirectional	^29–31^	Unknown (h)	Bidirectional	^31–33^	Lipoproteins, Amyloid-β, lactoferrin, ApoE
INSR	INSR	Unknown (h)	Blood to brain	^29,34^				Insulin
Tf-R	TFRC	Unknown (h)	Blood to brain	^29,35^	Unknown (h)	Blood to CSF	^35,36^	Fe-transferrin
FRα	FOLR1				Apical (h)	Blood to CSF	^37^	Folate
Aquaporin								
AQP-1	AQP1				Apical and basolateral (h)	Bidirectional; Net flow from blood to CSF	^38,39^	Water
ABC transporter								
*P-glycoprotein*								
P-gp (MDR1)	ABCB1	Luminal (h)	Endothelium to blood	^40,41^	Unknown (h), Apical (r)	Epithelium to CSF	^42^	Wide range of lipophilic, amphipathic molecules, steroids, phospholipids, bile acids; Size from 250 Da to 1850 Da
*Multidrug resistance proteins*								
MRP1	ABCC1	Luminal (h)	Endothelium to blood	^43^	Unknown (h), Basolateral (r)	Epithelium to blood	^42^	Lipophilic glutathione-, and sulphur-conjugates, more water-soluble, GSH, leukotriene C4, oestradiol, glucuronide
MRP2	ABCC2	Unknown (h), Luminal (r)	Endothelium to blood	^44,45^	Apical (h)	CSF to epithelium	^44^	Glucuronide and GSH conjugates, serine, alanine, arginine
MRP4	ABCC4	Luminal (h)	Endothelium to blood	^43^	Unknown (h), Basolateral (m)	Epithelium to blood	^36^	cAMP, cGMP, Bile acids, Steroids, } GSH, Prostaglandins
MRP5	ABCC5	Luminal (h)	Endothelium to blood	^43^				
*Breast cancer-related protein*								
BCRP	ABCG2	Luminal (h)	Endothelium to blood	^46^	Unknown (h), Apical (m)	Epithelium to CSF	^36,47^	Wide range of molecules, incl. sphingosine-1-P, bile acids, sulphate-conjugates, hypoxanthine, xanthine, glutamate
*Other*								
ABCA2	ABCA2	Unknown (h)	Unknown	^29^				Cholesterol
ABCA8	ABCA8	Unknown (h)	Unknown	^29^	Unknown (h)	Unknown	^36^	Taurocholate and estrone sulphate
SUR1	ABCC8	Unknown (h)	Bidirectional	^29,48^				Na^+^, K^+^
Solute carrier								
NKCC1	SLC12A2				Apical (h)	Bidirectional; Net flux from epithelium to CSF	^38,49^	Na^+^, K^+^, Cl^−^, Water
AE2	SLC4A2				Basolateral (h)	Bidirectional	^38^	Cl^−^, ${\text{HCO}}_{3}^{-}$
NCBE	SLC4A10				Basolateral (h)	Bidirectional	^38^	Cl^−^, ${\text{HCO}}_{3}^{-}$ , (natrium dependent)
NBCn1	SLC4A7				Basolateral (h)	Bidirectional	^38^	Na^+^, ${\text{HCO}}_{3}^{-}$
*Organic anion transporting polypeptides*								
OATP1C1	SLCO1C1	Unknown (h), Luminal and Abluminal (r)	Bidirectional	^50^	Mainly basolateral (h)	Bidirectional	^50^	Organic anion/bicarbonate exchangers (i.e., T4, rT3, conjugated and } unconjugated bile acids, prostaglandins, vasopressin)
OATP1A2	SLCO1A2	Luminal (h)	Bidirectional	^51,52^				
OATP2B1	SLCO2B1	Luminal (h)	Bidirectional	^51,52^				
OATP3A1	SLCO3A1				Apical and basolateral (h)	Bidirectional	^53^	
*Organic anion and cation transporter*								
OAT3	SLC22A8				Unknown (h), Apical (m)	Bidirectional; Net flux from CSF to epithelium	^36,54^	Dicarboxylate exchange with α-ketoglutarate, bicarbonate, Cl^−^
OCT1	SLC22A1	Luminal (h)	Bidirectional; Net flux from blood to endothelium	^55^				Organic cation/proton exchange } (incl. dopamine, adrenaline, serotonin, and choline)
OCT2	SLC22A2	Luminal (h)	Bidirectional; Net flux from blood to endothelium	^55^				
OCT3	SLC22A3	Unknown (h), Luminal and abluminal (r)	Bidirectional; Net flux from blood to brain	^56,57^				
OCTN1	SLC22A4	Unknown (h)	Unknown	^58^				Acetylcholine, acetyl-carnitine, carnitine, ergothioneine
OCTN2	SLC22A5	Unknown (h)	Unknown	^58^				Carnitine
URAT1	SLC22A12				Basolateral (h)	Bidirectional; Net flux from blood to epithelium	^59^	Uric acid
*Glucose transporter*								
GLUT1	SLC2A1	Unknown (h), Luminal and Abluminal (r)	Bidirectional; Net flux from blood to brain	^40,60^	Unknown (h), Basolateral (m)	Bidirectional; Net flux from blood to CSF	^36,61^	Glucose, Water
GLUT5	SLC2A5				Unknown (h), Apical (r)	Bidirectional; Net flux form epithelium to CSF	^36,62^	Fructose
GLUT9	SLC2A9				Apical (h)	Bidirectional; Net flux form epithelium to CSF	^59^	Urate
SGLT2	SLC5A2				Unknown (h)	Blood to epithelium	^63^	Glucose (sodium-dependent)
*Amino acid transporter*								
CAT1	SLC7A1	Unknown (h), Luminal and Abluminal (r)	Bidirectional; Net flux from blood to brain	^29,64^	Unknown (h)	Bidirectional	^36^	Basic L-amino acids: lysine, arginine (sodium-independent)
LAT1	SLC7A5	Unknown (h), Luminal and Abluminal (r)	Bidirectional; Net flux from blood to brain	^29,65^	Unknown (h)	Bidirectional	^36^	Asparagine, glutamate, histidine, isoleucine, leucine, methionine, phenylalanine, threonine, tryptophan, tyrosine, valine
ASCT1	SLC1A4	Unknown (h)	Unknown	^66^				Alanine, cysteine, glycine, isoleucine, } leucine, methionine, serine, threonine, valine (Sodium-dependent)
ASCT2	SLC1A5	Unknown (h)	Unknown	^66^				
EAAT1	SLC1A3	Unknown (h), Abluminal (b)	Bidirectional	^29,67^	Unknown (h)	Bidirectional	^36^	} Anionic amino acids Glutamate, } aspartate (Sodium-dependent), Water
EAAT2	SLC1A2	Unknown (h), Abluminal (b)	Bidirectional	^66,67^				
4F2HC	SLC3A2	Unknown (h)	Unknown	^29^	Unknown (h)	Unknown	^36^	Calcium, amino acids
*Nucleoside transporter*								
PMAT	SLC29A4	Luminal and abluminal (h)	Bidirectional; Net flux from brain to blood	^58^	Apical (h)	Bidirectional; Net flux from CSF to epithelium	^68^	Organic cation/proton exchange, serotonin, dopamine
ENT1	SLC29A1	Unknown (h)	Unknown	^29^	Unknown (h)	Unknown	^36^	Nucleosides, nucleotides, nucleobases
*Choline transporter*								
CTL1	SLC44A1	Unknown (h)	Unknown	^69^				} Choline
CTL2	SLC44A2	Unknown (h)	Unknown	^69^				
*Creatine transporter*								
CRT1	SLC6A8				Unknown (h)	Unknown	^36^	Creatine, Copper
*Organic cation transporter*								
MATE1	SLC47A1	Unknown (h)	Unknown	^58^	Unknown (h)	Unknown	^36^	} Peptides and nucleosides
MATE2	SLC47A2	Unknown (h)	Unknown	^58^				
*Fatty acid transporter*								
FATP1	SLC27A1	Unknown (h), abluminal (m)	Bidirectional	^66,70^				Long-chain fatty acids
*Folate transporter*								
RFC	SLC19A1	Unknown (h)	Bidirectional	^29^	Apical (h)	Bidirectional; Net flux from epithelium to CSF	^37^	Folate, thiamine derivatives
PCFT	SLC46A1				Unknown (h)	Unknown	^36^	Folate
*GABA transporter*								
BGT1	SLC6A12	Unknown (h)	Unknown	^29^	Unknown (h)	Unknown	^36^	γ-aminobutyric acid
*Monocarboxylic acid transporter*								
MCT1	SLC16A1	Unknown (h), Luminal and abluminal (r)	Bidirectional	^29,71^	Unknown (h)	Bidirectional	^36^	} Ketone bodies (incl. lactate, pyruvate)
MCT4	SLC16A4				Unknown (h)	Bidirectional	^36^	
MCT8	SLC16A2	Unknown (h), Luminal and Abluminal (r)	Bidirectional; Net flux from blood to brain	^50,66^	Unknown (h), Apical (r)	Bidirectional; Net flux from epithelium to CSF	^36,50^	T3 thyroid hormone (facilitative)

Transporters are only included when their presence has been detected using protein-based methods in human cells (see Methods). Of note, several transporters are strongly anticipated to be present at the human blood-brain of blood-CSF barrier, however, we found no evidence identifying their presence in human cells using protein-based methods. This includes, but is not limited to NKCC1 (SLC12A2, at the blood-brain barrier), Na^+^/K^+^-ATPase (ATP1A1), NHE1 (SLC9A1), NHE2 (SLC9A2), AE2 (SLC4A2, at the blood brain barrier), NBCe1 (SLC4A4), NBCn1 (SLC4A7, at the blood-brain barrier).

a Location in the endothelial cells of the blood-brain barrier: luminal (blood-facing), abluminal (brain-facing) or unknown (detected with a protein-based method at the blood brain-barrier, but cellular location unknown); protein confirmation in (h) = human cells, (r) = rat cells, (m) = mouse cells, (b) = bovine cells.

b Indicating transport direction of the molecules; blood to endothelium: from blood to the endothelial cells (without a confirmed role in transport from endothelial cells to brain). Blood to brain: from blood, across the endothelial cells of the blood-brain barrier, to the brain. Endothelium to brain: from the endothelium to the brain (without a confirmed role of transport from blood to endothelium). Bidirectional: transport in both directions likely across any of the barriers. Unknown = directionality unknown. Of note, especially solute carriers are capable of bidirectional transport dependent on concentration gradients. When the physiological most relevant direction is known, this is indicated.

c Location in the epithelial cells of the blood-CSF barrier: apical (CSF-facing), basolateral (facing endothelial cells) or unknown (detected with a protein-based method at the blood brain-barrier, but cellular location unknown); protein confirmation in (h) = human cells, (r) = rat cells, (m) = mouse cells, (b) = bovine cells.

d Indicating main direction of transport of the molecules; blood to epithelium: from blood to the epithelial cells (without a confirmed role in transport from epithelial cells to CSF). Blood to CSF: from blood, across the epithelial cells of the blood-CSF barrier, to the CSF. Epithelium to CSF: from the epithelial cells to the CSF (without a confirmed role of transport from blood to epithelium). Bidirectional: transport in both directions likely across any of the barriers. Unknown = direction unknown.

e Summarizing the main transported molecules including metabolites, proteins, water, vitamins, and electrolytes

## Results

To understand the basics of metabolomics of CSF to study brain metabolism, we reviewed evidence of how metabolites enter the CSF by transport across the BBB, BSCB or the BCSFB. Generally, transport mechanisms can be unidirectional or bidirectional using saturable transporter complexes or non-saturable mechanisms. In addition, transporters can be energy-dependent (movement of substrates against a concentration gradient) or deliver their substrates across the cellular membrane along their electrochemical gradient without energy consumption.^23,72^ As previously mentioned, all barriers contain tight junctions limiting paracellular diffusion; therefore, most metabolites enter or exit the CSF transcellularly. Carrier-mediated transporters at the BBB can be located at the luminal side (blood-facing) or the abluminal side (facing brain parenchyma) of the endothelial cells or both. Likewise, at the BCSFB transporters can be located apically (CSF-facing) and at the basolateral side (blood-facing) of the epithelial cells of the choroid plexus.

**Figure 1.**
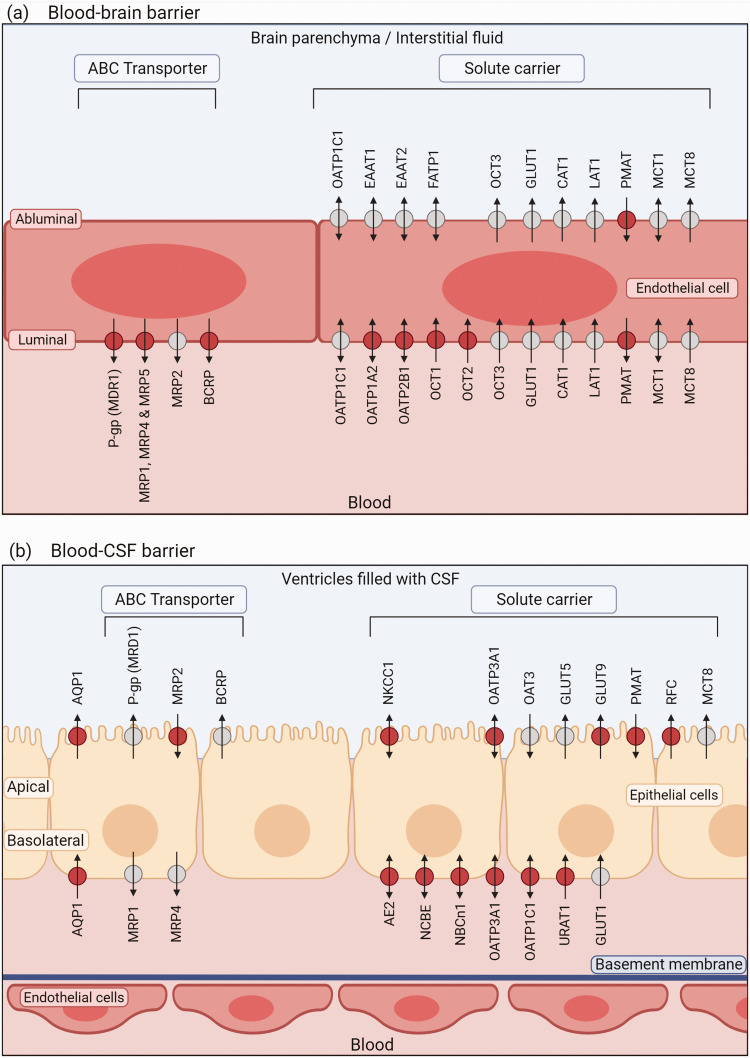
Transporters at the blood-CSF barrier and blood-brain barrier. Simplified illustration of the blood-brain barrier without the adjacent basement membrane, pericytes and astrocyte end feet located at the abluminal endothelial side. Only transporters of which the exact cellular location is known are depicted in the figure. Red circles indicate transporters of which location was demonstrated in human cells; grey circles indicate the transporter location was shown in animal cells. Arrows indicate the presumed main transport direction. In case of solute carriers capable of bidirectional transport, the physiological most relevant direction is indicated. Of note, the following transporters are present at the barriers but not included in the figure because their cellular location has not yet been shown: OCTN1, OCTN2, ASCT1, ASCT2, 4F2HC, ENT1, CTL1, CTL2, MATE1, MATE2, RFC, BGT1, ABCA2, ABCA8, SUR1 for the blood-brain barrier and ABCA8, SGLT2, CAT1, LAT1, EAAT1, 4F2HC, ENT1, CRT1, MATE1, PCFT, BGT1, MCT1 MCT4 for the blood-CSF barrier. Image created in BioRender.com.

Many molecules are transported by two different transporter superfamilies: The ATP-binding cassette (ABC) superfamily and the solute carrier (SLC) superfamily. Generally, ABC transporters function as primary active efflux transporters, moving their substrates out of endothelial cells into the bloodstream or CSF by using metabolic energy (ATP hydrolysis). SLC proteins mostly facilitate the uptake of their substrates into cells, predominantly passively/facilitative or secondary (i.e. without direct ATP involvement) active, thereby removing various molecules from the CSF and bloodstream.^73^ A detailed overview of the transporters, their cellular location, and their directionality can be found in Table 1 and Figure 1.

### Passive diffusion

Only small lipophilic metabolites, as well as small gaseous molecules like O_2_ or CO_2_, can freely diffuse transcellularly across the lipid membranes of the epithelial and endothelial cells of all three barriers following their concentration gradient.^10,74^ However, diffusion depends highly on the lipid solubility, enabling small hydrophobic metabolites like caffeine, and nicotine to enter the CSF but restraining the entrance of larger lipophilic metabolites.^72,75^ Other properties restricting the diffusion of metabolites across all CNS barriers include a high polar surface area, rotatable bonds, and a high affinity to plasma proteins.^76,77^

### Vesicular transport

Vesicular transport regulates transport of a limited group of macromolecule proteins, and some metabolites into the CSF. In general, this process involves micro-invagination of the outer membrane, which is then pinched off as a vesicle, migrates with its cargo across the cell, and fuses with the membrane to release its content.^78^ Three forms of vesicular transport play a role in CNS transport: fluid-phase transcytosis is the non-specific uptake of the interstitial fluid by cells transporting limited amounts of transferrin^79^ and immunoglobin G^80^ across the BBB; absorptive-mediated endocytosis, facilitates the transport of cationic molecules like cationized albumin by binding to negatively charged membrane surface molecules;.^78^ Lastly, receptor-mediated transcytosis is important for entrance of transferrin^81^ and insulin low-density lipoproteins^29,36^ across the BBB and folate across the BCSFB into the CSF. Folate receptor-α (FRα) facilitating receptor-mediated transport has been shown to transport folate from the basolateral membrane to the apical membrane where most receptors accumulate. FRα-receptor deficiency is indeed associated with low folate CSF concentrations.^37^

### Aquaporins

Water homeostasis in the CSF and blood must be tightly regulated to prevent disturbances leading to deleterious effects like brain edema.^82^ Aquaporins (AQP) are molecular water channels expressed by various tissues, including the CNS. In the epithelial cells of the human choroid plexus (BCSFB), AQP-1 is expressed more abundantly in the apical than the basolateral membranes,^38^ serving as a rate limiting factor for water movement.^83,84^ AQP-1 allows bidirectional transport, with an inward net flux. It has been suggested that AQP-1 functions as an osmosensor that can adjust the water transport rates based on the molarity of the CSF.^85^ AQP4, present at astrocytic but not glial end feet, may be important to water transport at the BBB and in the glymphatic system.^86,87^

### ABC transporters

ATP-binding cassette (ABC) transporters, also called efflux pumps, are known as drug transporters and transport their substrates out of the cells. ABC transporters belong to the largest and evolutionarily oldest superfamily highly conserved across species. The ABC transporter superfamily is organized into seven families, of which three (i.e., ABCB, ABCC and ABCG) have a known role in the human CNS and are discussed below in more detail. Research has mostly focussed on their role in drug transport, and their metabolite specificity is less clear. However, a study measuring metabolite levels using mass spectrometry following efflux transporter inhibition in colon carcinoma cells, indicates increasing intracellular levels of eleven metabolites including glutamine, phenylalanine, threonine, and methionine with inhibition of ABC transporter P-glycoprotein (ABCB1). Moreover, decreasing xanthine, hypoxanthine, and glutamate levels were found with inhibition of breast cancer-related protein (BCRP or ABCG2) and increasing concentration of serine, alanine, arginine, and other methionine metabolites with inhibition of multidrug resistance protein 2 (MRP2 or ABCC2).

#### P-glycoprotein

The multidrug resistance gene product 1 p-glycoprotein (MDR1, P-gp, ABCB1) is a prominent ABC transporter. Its overexpression in tumour cells was discovered to enhance multidrug resistance, hence its name.^88^ P-gp is N-glycosylated, comprising transmembrane domains and intracellular ATP-binding sites utilizing hydrolysis to pump substrates against their concentration gradient,^89^ mostly as an efflux transporter pumping its substrates out of cells. Substrates range from 250 Da to 1850 Da in molecular weight and include a wide range of proteins and metabolites that are generally lipophilic and amphipathic, with the highest transport efficiency for basic or uncharged molecules. In homeostatic circumstances ABC transporter knockout mice showed no physiological abnormalities,^90^ indicating substrate overlap with other transporters. In the CNS, P-gp is located primarily in the luminal membrane of endothelial cells of the BBB,^40^ where it pumps its substrates into the blood. It is also located in the epithelial cells of the choroid plexus (BCSFB) in humans, rats, and mice. In rodents, it was found in higher abundance at the apical membrane, facilitating active transport from blood into the CSF.^42,91^

#### Multidrug resistance proteins

Another family of ABC transporters is the multidrug resistance protein (MRP or ABCC) family. At the BBB they have large substrate overlap with other ABC transporters, facilitating the efflux of various metabolites and drugs. Endogenous substrates include, but are not limited to, lipophilic glutathione- and sulphur-conjugates.^43,91^ Knockout studies in mice showed that similar to the P-gp knockout mice, the MRP1 and MRP4 knockout animals have normal physiology but show significantly increased drug uptake into the brain.^19^ MRP4 has been found at the basolateral membrane of the epithelial cells of the choroid plexus transporting its substrates out of the CSF into the bloodstream.^19,92^ This has been confirmed by experiments in mice showing that MRP4 knockout animals had significantly higher concentrations of topotecan, an anti-cancer drug, in the CSF and brain compared to wild-type mice.^92^ In addition, MRP1 also has been found at the basolateral membrane of the choroid plexus in humans, where it increases the efflux of its substrates from the CSF into the blood, opposing the efflux into the CSF generated by P-gp.^93^ Due to an overlap in substrate specificity, substrate concentrations may also depend on the interplay between different receptors and their relative concentrations in the different membranes of the BBB and the BCSFB.

Sulfonylurea receptor 1 (SUR1 or ABCC8) is located in brain endothelial cells and can form an ATP sensitive potassium channel (K_ATP_) with Kir6.1 and Kir6.2 subunits.^94^ Moreover, SUR1 is upregulated after cerebral infarcts and forms a complex with the transient receptor potential melastatin 4 (Trpm4). This SUR1-Trpm4 complex leads to increased influx of sodium into endothelial cells causing swelling and ultimately BBB disruption.^48^

#### Breast cancer-related protein

Breast cancer-related protein (BCRP, ABCG2) is another transporter of the ABC superfamily which is highly abundant in several tissues including the BBB. Its substrates largely overlap with those of P-gp and both transporters work in concert limiting the access of various metabolites and drugs to the brain.^95^ In line with its function as an efflux transporter, BCRP has been located at the luminal surface of BBB endothelial cells.^46^ Its cellular location at the human choroid plexus however is less clear. A previous study identified the presence of BCRP at the choroid plexus using quantitative targeted proteomics.^36^ Controversially, in another study using immunohistochemistry, no signal was detected in the epithelial cells of the human choroid plexus.^44^ In mice choroid plexus, BCRP has been detected at the apical surface facing the CSF.^47^

### Solute carriers (SLC)

The superfamily of SLC is a highly diverse group of transport proteins with relatively narrow specificity. This includes 52 families transporting for example amino acids, glucose and organic anions and cations.^96^ Most of the transporters can facilitate bi-directional transport, but given the osmotic gradient, they are mainly responsible for their substrates’ net cellular uptake.^73^ A wide variety of ion transporters belonging to the solute carrier superfamily are located at the choroid plexus epithelium to ensure CSF production and cellular ion homeostasis. Some transporters facilitate the exchange of their substrates by directly or indirectly utilizing ion gradients generated by ion pumps like the Na^+^-K^+^ ATPase.^73^ Located at the apical surface of the epithelial cells of the choroid plexus, Na^+^-K^+^ ATPase continuously pumps Na^+^ into the CSF, creating an osmotic gradient which leads to increased water diffusion into the ventricular lumen.^38^ Interestingly, the distribution of sodium transporters and Cl^−^ ${\text{HCO}}_{3}^{-}$ exchangers within the human choroid plexus is almost identical to the structure of the choroid plexus of mice or rats, indicating that those animals could be suitable model organisms to study sodium exchange in the choroid plexus.^38^

#### Organic anion transporting polypeptides

The organic anion-transporting polypeptides (OATPs, SLCO) subfamily of the solute carriers include mainly sodium-dependent and -independent carriers. These transmembrane proteins contain 12 transmembrane domains facilitating bi-directional exchange with a broad range of relatively large substrates containing both polar and non-polar groups (amphipathic) like steroids, thyroid hormones, and bile salts. Uptake of organic anions from the CSF or the blood occurs at the apical and luminal membrane via exchange with intracellular glutathione or ${\text{HCO}}_{3}^{-}$ .^97^

#### Organic anion and cation transporter

The SLC22 subfamily contains multi-specific organic anion transporters (OAT) and organic cation transporters (OCT), transporting smaller, hydrophobic anions and cations. Both transporter families are known to facilitate the transport of endogenous substrates and various xenobiotics like pesticides, herbicides, and drugs. Endogenous substrates of OATs include especially small neurotransmitters like cAMP, cGMP, and some prostaglandins. Transport of those substrates is facilitated by the exchange of dicarboxylate, the reuptake of which is coupled to sodium transport utilizing the sodium gradient maintained by the Na^+^-K^+^ ATPase.^73^ OCTs transport their substrates mainly utilizing the membrane potential and concentration gradients of their substrates, but for some subtypes, active sodium or proton-coupled transport has also been observed. Their substrates include, among others, epinephrine, histamine, choline, and carnitine.^98^

#### Glucose transporter

The continuous utilization of glucose by the brain creates a concentration gradient to transport glucose from the blood into the brain and into CSF across the choroid plexus.^74^ Two main glucose transporter families, maintain sufficient glucose supply to the brain. The insulin insensitive GLUT1 transporter (SLC2A1), located both at the choroid plexus epithelial cells and BBB endothelial cells is the most important, providing net transport into brain and CSF, with also an important reverse flux. Additionally, SGLT2 (SLC5A2), a low-affinity sodium-dependent glucose transporter, has been shown to be present in the choroid plexus of human and mouse brains.^63^

#### Amino acid transporter

Amino acids are essential for protein synthesis and repair and are therefore needed for the brain’s functioning. For the synthesis of neurotransmitters like histamine, serotonin, and dopamine, essential amino acids extracted from the diet need to be taken up via the circulation. Within the BBB, various amino acid carriers are found (mostly SLC7A, SLC1A and SLC3A), allowing the transport of these polar metabolites into the brain. The carriers can be found at the apical and basolateral membranes of the endothelial cells of the BBB but also within the membranes of the epithelial cells of the choroid plexus.^99^ A net influx of amino acids through the BCSFB in the choroid plexus has been demonstrated in sheep, but the resulting CSF concentrations remain lower than those in blood. Most amino acid carriers are sodium-dependent co-transporters driven by a sodium gradient. Besides fulfilling the brain’s amino acid requirements, some carriers also transport excitatory neurotransmitters such as glutamate and aspartate out of the brain parenchyma.^78,99^

#### Active water transport

It has long been believed that CSF production resulted from passive water transport through aquaporins into the lumen of the ventricles, following active ion transport. However, the observed osmotic difference of 5 mOsm fails to explain how epithelial cells of the choroid plexus secrete CSF at a high rate along this relatively weak osmotic gradient.^100^ To explain the observed CSF secretion rate, the calculated osmotic difference between the plasma and the CSF should have been at least 250 mOsm.^83,100^ Experiments using AQP-1 knockout mice demonstrated only a 20% reduction in CSF production and this might even be partly explained by reduced blood pressure.^84^ Therefore, besides AQP-1, there needs to be another mode of entry for water. Indeed, many co-transporters involve transport of water with different ions or metabolites, including the glucose transporter GLUT1 (SLC2A1), glial glutamate transporter EAAT1 (SLC1A3), Na^+^-K^+^-2Cl^−^ co-transporter NKCC1, and K^+^-Cl^−^ co-transporter KCC1 (SLC12A4).^100,101^ The last two were found in the epithelium of the choroid plexus of humans and mice, respectively. Of note, NKCC1 maintains a high CSF production rate independent of an osmotic gradient.^84^

## Discussion

Metabolomics of the CSF is used to better understand brain metabolism in health and disease. Interpreting CSF findings should consider metabolite entry to and exit from the CSF. We therefore provide an overview of transporters on the apical and basolateral sides of the BCSFB transporting directly into or out of the CSF and of the luminal and abluminal sides of the BBB transporting into or out of the brain. Once brain cells have used metabolites, they are removed via the BBB into the circulation and via interstitial fluid exchange to the CSF. Many different amino acids, nucleotides, fatty acids, and glucose are transported across the three barriers. Consequently, CSF metabolite levels represent the net result of influx, use by the brain and efflux.

Interpretation of CSF metabolite levels need to consider several factors. First, CSF samples are usually taken via lumbar puncture which is anatomically remote from the brain parenchyma. Constant influx and efflux of metabolites along the neuroaxis could lead to altered metabolite abundances in lumbar CSF explaining the previously observed rostrocaudal concentration gradient of several metabolites including homovanillic acid and 5‐hydroxyindoleacetic acid.^8^ In comparison to the lumbar CSF, ventricular CSF has lower protein content, a higher chloride concentration and a higher CSF/blood glucose ratio.^102^ Analogously to modelling studies on CNS drugs and drug-like molecules,^75,76^ CSF metabolite studies could potentially use in silico approaches to account for the penetration ability of different metabolites across the different CNS barriers along the neuroaxis to correct for this when the goal is to predict metabolite concentrations in the brain.

Increased permeability of the brain barriers during inflammation further influences the interpretation of metabolomics studies, leading to an increase of directly blood-derived metabolites in the CSF of patients. Additional to this leakage, a reduced CSF flow prolonging the time for exchange along the neuroaxis, might also explain the altered metabolite and protein content in lumbar CSF with an increasing fraction of blood-derived in the lumbar CSF of patients with more inflammation.^103^ For example, in tuberculous meningitis patients, who are known to have prolific inflammation with consequent disruptions to CSF flow,^104^ 70% of the measured metabolites where higher than in controls.^5^ This increase in CSF metabolites in highly inflammatory conditions may thus be partially explained by this increased blood-fraction in addition to an increase in central nervous system metabolism or brain damage.^5^ A few studies have designed microfluidic organ-on-chip models for the BBB which could be used in the future to understand how changes in permeability influence metabolite concentrations.^105^

Limitations of this review include the *in vitro* source of most of the knowledge on the precise location of the transporters presented in Table 1. The culture conditions might influence the availability of the transporters, i.e. one study showed the increased expression of GLUT1 and P-gp in endothelial membranes in the presence of co-cultured astrocytes compared to culturing endothelial cells alone revealing the importance of signals from other cell types.^106^ By restricting to transporters for which the location was proven by protein-based methods rather than transcription-based methods, we increased specificity, but this precluded us from including transporters demonstrated only at a transcriptional level. Furthermore, although channel distribution in murine and human choroid plexus cells are similar,^38^ another limitation is that rodents were used for the *in vivo* studies in 38% (12/31) of the presented studies. Using animal knockout models for transporters not exclusively expressed at the BCSFB or the BBB, can be biased by induced alterations in non-CNS compartments. Lastly, *in vitro* transporter knock-outs may be compensated by upregulation of another transporter.^19^ Moreover, for many drug transporters,^107^ possible metabolite substrates are not yet known. Despite these limitations, this manuscript is the first attempt to evaluate influx and efflux routes of metabolites in the CNS considering factors influencing metabolite concentrations in the CSF. We identified uncertainties accompanying metabolomics of the CSF which have previously largely been neglected. Moreover, we provide an extensive collection of the transporters and receptors facilitating metabolite transport across the human BBB and BCSFB.

In conclusion, CSF metabolomics provides unique opportunities to study the CNS metabolome, in which the constant production and reabsorption of metabolites along the central nervous system (CNS) needs to be considered. This review emphasizes the importance of the underlying physiology when interpreting CSF findings, and specifically (1) the involved transporters on the blood-CSF barrier and their direction of transport, (2) the contribution of passive diffusion directly of blood-derived metabolites into the CSF, especially with increased permeability such as occurs in inflammatory conditions, (3) the realization that lumbar CSF is further away from the central metabolism than the ventricular CSF. The increased availability of publicly available data on metabolite levels and transporter expression levels, will help our interpretation of CSF metabolomic finding, leading to better understanding of brain metabolism.
